# Design and Analysis of the Measurement Characteristics of a Bidirectional-Decoupling Over-Constrained Six-Dimensional Parallel-Mechanism Force Sensor

**DOI:** 10.3390/s17091985

**Published:** 2017-08-30

**Authors:** Zhi Niu, Tieshi Zhao, Yanzhi Zhao, Qiangqiang Hu, Shixing Ding

**Affiliations:** 1 Key Laboratory of Parallel Robot and Mechatronic System of Hebei Province, Yanshan University, Qinhuangdao 066004, China; zniu@stumail.ysu.edu.cn (Z.N.); yzzhao@ysu.edu.cn (Y.Z.); qianghu1993@126.com (Q.H.); dingshixing@stumail.ysu.edu.cn (S.D.); 2 Key Laboratory of Advanced Forging & Stamping Technology and Science of Ministry of Education of China, Yanshan University, Qinhuangdao 066004, China

**Keywords:** force sensing, load decoupling, steel ball structure, over-constrained sensors, six-dimensional sensors, parallel mechanism

## Abstract

The measurement of large forces and the presence of errors due to dimensional coupling are significant challenges for multi-dimensional force sensors. To address these challenges, this paper proposes an over-constrained six-dimensional force sensor based on a parallel mechanism of steel ball structures as a measurement module. The steel ball structure can be subject to rolling friction instead of sliding friction, thus reducing the influence of friction. However, because the structure can only withstand unidirectional pressure, the application of steel balls in a six-dimensional force sensor is difficult. Accordingly, a new design of the sensor measurement structure was designed in this study. The static equilibrium and displacement compatibility equations of the sensor prototype’s over-constrained structure were established to obtain the transformation function, from which the forces in the measurement branches of the proposed sensor were then analytically derived. The sensor’s measurement characteristics were then analysed through numerical examples. Finally, these measurement characteristics were confirmed through calibration and application experiments. The measurement accuracy of the proposed sensor was determined to be 1.28%, with a maximum coupling error of 1.98%, indicating that the proposed sensor successfully overcomes the issues related to steel ball structures and provides sufficient accuracy.

## 1. Introduction

As industries such as aerospace, human body biomechanics measurement, and mechanical processing continue to push the envelope of scientific capability, the accuracy and decoupling of sensing systems has grown in importance, particularly considering the increased interest in manned space expeditions. These applications require the ability to not only measure heavy loads, but also sense the full six-dimensional range of force information (magnitude, direction, and point of application). Various researchers have already demonstrated the successful use of multi-dimensional force/torque sensors constructed from multi-component structures. For example, Watson et al. developed a three vertical-rib structure for a six-dimensional force sensor [1]. Kroll developed a six-axis sensor for measuring robot wrist force [2]. Gao et al. used elastic ball joints instead of spherical joints to create a Stewart parallel-structure six-dimensional force sensor, an innovation that allowed for the miniaturisation of the parallel-mechanism structure sensor [3,4].

With the rapid development of aerospace capabilities, the need to measure heavy loads has become a significant challenge for the six-dimensional force sensor. Parallel mechanisms have considerable advantages, such as high stiffness in conjunction with high sensitivity and measurement isotropy, for use in six-dimensional force sensors [5]. Hiroserk et al. used six single-dimensional force sensors to form a six-dimensional force sensor based on the Stewart parallel structure [6]. Ranganath et al. developed a new non-singular version of the Stewart platform six-dimensional force sensor [7]. Dwarakanath developed a six-dimensional force sensor based on the Stewart parallel structure, and performed structural optimization [8,9]. Wright et al. invented a six degree-of-freedom thrust sensor for a lab-scale hybrid rocket [10]. Wang et al. developed two kinds of pre-stressed six-axis force sensors, and analysed their accuracy and dynamic responses [11,12]. Further, Jia et al. developed a wide range of sensors based on the Stewart structure [13]. Yao et al. proposed a task-oriented method for a six-axis force sensor based on the Stewart platform [14]. By examining the current common configurations of parallel six-dimensional force sensors, it is clear that the addition of a redundant measurement branch can enhance the measurement range of a six-dimensional force sensor.

However, adding redundant measurement branches to a parallel six-dimensional force sensor results in an over-constrained structure. In addition, the measurement model of a typical parallel six-dimensional force sensor is different from that of an over-constrained six-dimensional force sensor. In this vein, Pashikevich [15] proposed a stiffness modelling method based on branch nodes for over-constrained parallel structures. Janus et al. analysed the dynamic of an over-constrained mechanism by considering joint friction [16]. Choi et al. analysed the static and dynamic models of an over-constrained parallel mechanism [17]. Yao et al. studied the static-indeterminate mapping and fault tolerance performance of a redundant-structure six-axis force sensor [18,19]. 

It is important to note that when the load on a six-dimensional force sensor is increased, the friction between the branches and couplings will also increase. For a parallel six-dimensional force sensor under heavy loads, the need to enhance the sensor range while effectively reducing the dimensional coupling poses a significant challenge in the sensor’s development. The adoption of a new measurement structure is widely accepted as an effective method for reducing dimension coupling. As a result, research to this effect has been conducted by many scholars. Dwarakanath et al. proposed a six-dimensional force sensor with a new measurement branch, and the proposed sensor was demonstrated to possess superior isotropy and sensitivity over a conventional sensor configuration [20]. Zhao et al. proposed a conic sphere pair to reduce dimensional coupling [21]. Liang et al. designed a new six-dimensional force sensor by using elastic elements in the measurement branch to reduce dimensional coupling, and applied it for measuring the cutting force in machining processes [22,23]. In addition, Zhao et al. proposed a six-dimensional force sensor, in which flexible joints with large measurement ranges were adopted to reduce dimensional coupling [24]. Lu et al. designed and analysed a novel force/torque sensor for a hybrid hand with three fingers [25]. Kim et al. presented a novel six-axis force/torque sensor for robotic applications, and a sensor design with parallel and orthogonal arrangements of sensing cells; this was found to achieve a large improvement in sensitivity [26]. Finally, Yang proposed a novel three-dimensional force sensor based on a parallel mechanism, demonstrating the proposed sensor to be more sensitive to shear forces than to normal forces [27].

The use of a steel ball structure instead of a conventional ball pair as a measuring branch is an effective method for reducing dimensional coupling caused by friction. Zhao et al. designed a three-dimensional force sensor by using a steel ball structure to decouple the dimensional coupling [28]. However, a significant issue arises with the use of steel ball structures in sensors: the steel ball can only accommodate pressure in the measurement direction; thus, when it is subjected to tension, the ball separates from the contact surface. This implies that the steel ball structure can only be used in force sensors capable of measuring a limited number of dimensions and it is a difficult mechanism to apply in a six-dimensional force sensor. Therefore, the use of a steel ball structure in a six-dimensional force sensor, which promises high load capacity and reduced dimension coupling, remains a problem that must be solved.

Based on this clear need, this paper proposes an over-constrained six-dimensional parallel mechanism force sensor using steel ball structures to reduce dimensional coupling caused by friction. This approach requires the design of a new branch structure such that the branch can accommodate both tension and pressure while remaining compact. However, as the proposed sensor has an over-constrained rather than a statically determinate structure, the force distribution is different from that of a typical six-dimensional sensor. Accordingly, this study established a new measurement model based on the proposed six-dimensional force sensor configuration; the force in the measurement branches were analytically derived, and the measurement characteristics of the sensor were then analysed through numerical examples. A calibration experiment was then conducted and verified by a sensor application experiment, thus obtaining the measurement accuracy of the sensor.

The remainder of this paper is organised as follows: Section 2 describes the structure and measurement principle of the proposed six-dimensional force sensor. Section 3 derives the transformation function of the proposed sensor. Section 4 discusses the numerical simulation results for the calculated model of the proposed sensor. Section 5 presents the results of the sensor calibration and application experiments. Finally, Section 6 presents some brief concluding remarks, summarizing the work.

## 2. Sensor Structure and Measurement Principle

### 2.1. Sensor Structure

**Figure 1:**
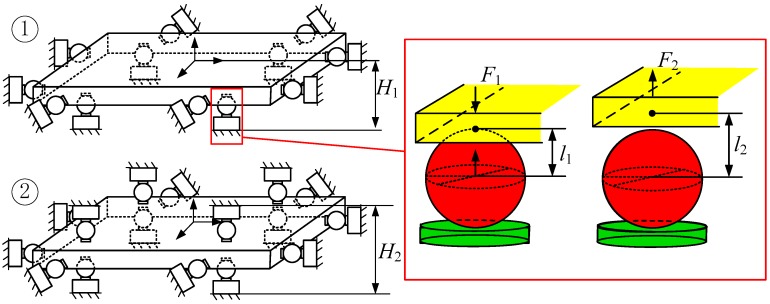
Schematic of a steel ball structure multi-dimensional force sensor.

The steel ball structure used in the proposed six-dimensional force sensor measuring branch is subject to rolling friction instead of sliding friction, thus reducing the sensor’s dimensional coupling. However, the steel ball structure can only withstand pressure in the measuring direction; when the steel ball is subjected to tension, the ball separates from the contact area. As shown in Figure 1, when the contact part (yellow) is subjected to a downward force *F*_1_, the steel ball structure (red) will experience the reacting force; however, when upward tension *F*_2_ is applied, the contact part will pull away from the steel ball, and the distance between the centre of the ball and contact part increases from *l*_1_ to *l*_2_. One way to solve this problem is to arrange the susceptible measuring branches in pairs around the force plate (as shown in Image 2 in Figure 1), resulting in a more complex and less compact structure in which the distance between the two measuring modules is increased from *H*_1_ (in Image 1) to *H*_2_ (in Image 2). However, for many potential six-dimensional force sensor applications, installation space is limited; thus, it is important that the sensor structure be both simple and compact.

Considering these limitations, the combined use of horizontal and vertical measurement modules is proposed in this study. Each of these modules uses a steel ball structure, and thus benefits from lower friction, while simultaneously allowing for the measurement of both pressure and tension in their given orientation.

**Figure 2:**
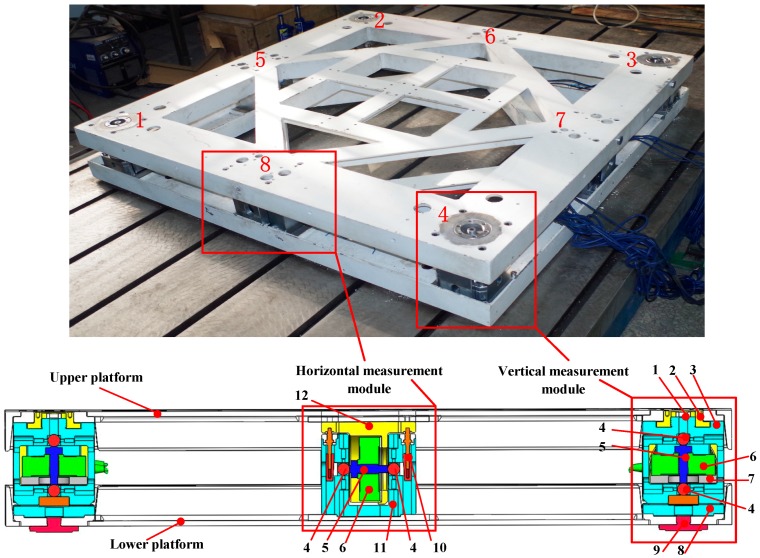
Proposed bidirectional-decoupling over-constrained six-dimensional force sensor.

Four vertical (1–4) and four horizontal (5–8) measurement modules were assembled in the appropriate orientations to construct the proposed bidirectional-decoupling over-constrained six-dimensional force sensor, shown in Figure 2.

Table 1 lists the labelled parts in Figure 2.

Spoke-type force sensors are installed one each on the lower vertical and upper horizontal branch modules. Note that there are steel ball structures one each at the top and bottom of the upper vertical branch module and on the left and right sides of the lower horizontal branch module. The adjustment component indicated in the figure was used to adjust the contact between the vertical measurement modules and the upper platform. The adjustment part was used to adjust the height of the sensor. Positioning devices were arranged around the steel balls, and were used to adjust the position of the steel ball so that it was concentric with the spoke-type force sensor. A pre-tightening device was used to adjust the clearance to ensure good contact between parts. Table 2 lists the specifications of the major parts in Table 1.

The spoke-type force sensor also has the following specifications: (1) rated output: 3.0 m *V*/*V*; (2) repeatability: ±0.03 of rated output; (3) non-linearity: ±0.03 of rated output; (4) excitation voltage: 10 V, DC/AC.

### 2.2. Sensor Measurement Principle

**Figure 3:**
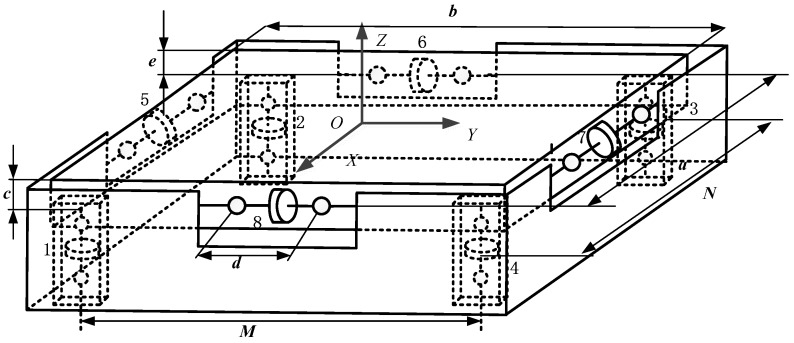
Schematic of the proposed over-constrained six-dimensional force sensor.

Figure 3 shows the schematic of the proposed sensor. Based on screw theory, the measuring principle can be expressed as:

(1) F=Gf

where ***F*** is the generalized external force, ***f*** is the measuring force vector of the branch, and ***G*** is the geometrically derived force-mapping matrix such that:

(2) $$G=\left[\begin{array}{cccc} S_{1} & S_{2} & \ldots & S_{8}\\ S_{O1} & S_{O2} & \ldots & S_{O8} \end{array}\right]$$

where ***S****_i_* represents the unit line vector along the *i*-th measuring direction, which yields the following matrix by using the parameters of the sensor shown in Figure 3:

(3) $$G=\left[\begin{array}{cccccccc} 0 & 0 & 0 & 0 & 1 & 0 & 1 & 1\\ 0 & 0 & 0 & 0 & 0 & 1 & 0 & 1\\ 1 & 1 & 1 & 1 & 0 & 0 & 0 & 0\\ -\frac{M}{2} & -\frac{M}{2} & \frac{M}{2} & \frac{M}{2} & 0 & \frac{e}{2} & 0 & \frac{e}{2}\\ -\frac{N}{2} & \frac{N}{2} & \frac{N}{2} & -\frac{N}{2} & -\frac{e}{2} & 0 & -\frac{e}{2} & 0\\ 0 & 0 & 0 & 0 & \frac{b}{2} & -\frac{a}{2} & -\frac{b}{2} & \frac{a}{2} \end{array}\right]$$

## 3. Sensor Measurement Model

### 3.1. Derivation of the Compatibility Equation

For over-constrained structures, the interaction between the stiffnesses of each component results in changes in the internal force distribution. Therefore, to establish a measurement model for the proposed over-constrained six-dimensional force sensor, the different stiffnesses of each branch must be considered.

**Figure 4:**
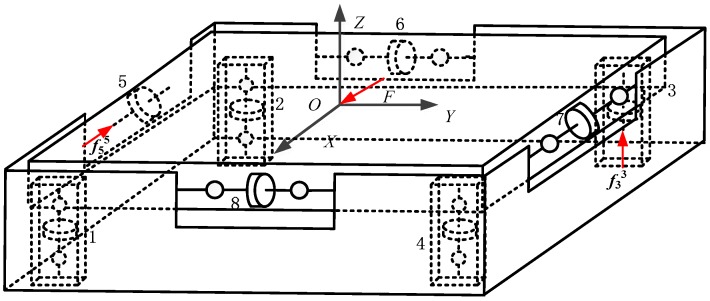
Force diagram of the basic system of the proposed sensor.

The proposed sensor has eight force branches, as shown in Figure 4. According to the force method of structural mechanics and static equilibrium equation, the measurement modules indicated by Labels 3 and 5 are the redundant constraints, and are thus replaced by unit forces *f*_3_^3^ and *f*_5_^5^, respectively, indicated by the red arrows in Figure 4.

The compatibility equation for these forces is:

(4) $$\begin{array}{l} \delta_{33}f_{3}+\delta_{35}f_{5}+\Delta_{3F}=0\\ \delta_{53}f_{3}+\delta_{55}f_{5}+\Delta_{5F}=0 \end{array}$$

where *f*_3_ and *f*_5_ are the internal forces for measurement Modules 3 and 5, respectively, when the sensor is under the generalized external force *F*; $\delta_{ij}$ is the deformation along the *i*-th module when the force *f_j_^j^* is used to replace the redundant constraint in the basic system; $\Delta_{iF}$ is the deformation along the *i*-th module when the basic system is under the generalized external force *F*. The values of $\delta_{i_{1}i_{2}}$ and $\Delta_{iF}$ can be obtained by:

(5) $$\delta_{i_{1}i_{2}}=\sum_{j=1}^{8}\frac{{f_{j}}^{i_{1}}{f_{j}}^{i_{2}}l_{j}}{E_{j}A_{j}}\;(i_{1}=3,5,\;i_{2}=3,5),\;\Delta_{iF}=\sum_{j=1}^{8}\frac{{f_{j}}^{i}{f_{j}}^{t}l_{j}}{E_{j}A_{j}}\;\left(i=3,5\right)$$

where *l_j_* is the length of the *j*-th module, *E_j_* is the elastic modulus, *A_j_* is the cross-sectional area of the steel ball, and *f_j_^t^* is the internal force on each module when the basic system is subjected to external force *F.*

The forces *f_j_^i^* and *f_j_^t^* can be represented as the matrices:

(6) $$f_{\delta }=\left[\begin{array}{cc} {f_{1}}^{3} & {f_{1}}^{5}\\ {f_{2}}^{3} & {f_{1}}^{5}\\ \vdots & \vdots \\ {f_{8}}^{3} & {f_{8}}^{5} \end{array}\right],\;f_{\Delta }=\left[\begin{array}{c} {f_{1}}^{t}\\ {f_{2}}^{t}\\ \vdots \\ {f_{8}}^{t} \end{array}\right]$$

respectively, where *f_j_^i^* and *f_j_^t^* can be classified into the following cases, as summarized in Table 3: (1) *f_j_^i^* is the force on measurement Modules 3 and 5 where *i* = *j*, in which *f_j_^j^* is used to replace the redundant constraint in the basic system; (2) *f_j_^i^* is the force on the measurement Modules 3 and 5 where *i* ≠ *j*, in which *f_j_^j^* is used to replace the redundant constraint in the basic system; (3) *f_j_^i^* is the force on measurement modules other than 3 and 5, in which *f_j_^j^* is used to replace the redundant constraint in the basic system; (4) *f_j_^i^* is the force on measurement modules 3 and 5, in which the basic system is under the generalized external force; (5) *f_j_^i^* is the force on the measurement modules other than 3 and 5, in which the basic system is under the generalized external force.

From Equation (2):

(7) $$G_{I}=\left[\begin{array}{ccccc} S_{1} & \cdots & S_{i} & \cdots & S_{8}\\ {S_{O}}_{1} & \cdots & {S_{O}}_{i} & \cdots & {S_{O}}_{8} \end{array}\right],\;i\neq 3,5$$

and by using Table 3, the force *f_k_* can be expressed as:

(8) $$f_{k}=\left[\begin{array}{ccc} {f_{3}}^{3} & {f_{3}}^{5} & {f_{3}}^{t}\\ {f_{5}}^{3} & {f_{5}}^{5} & {f_{5}}^{t}\\ {f_{1}}^{3} & {f_{1}}^{5} & {f_{1}}^{t}\\ \vdots & \vdots & \vdots \\ {f_{8}}^{3} & {f_{8}}^{5} & {f_{8}}^{t} \end{array}\right]=\left[\begin{array}{ccc} 1 & 0 & 0\\ 0 & 1 & 0\\ {G_{I}}^{-1}G_{3} & {G_{I}}^{-1}G_{5} & {G_{I}}^{-1}F \end{array}\right]=\left[\begin{array}{cc} I & 0\\ {G_{I}}^{-1}G_{\mathrm{II}} & {G_{I}}^{-1}F \end{array}\right]$$

where, again, from Equation (2):

(9) $$G_{\mathrm{II}}=\left[\begin{array}{cc} S_{3} & S_{5}\\ {S_{O}}_{3} & S_{O5} \end{array}\right]$$

Equation (8) can then be substituted into the compatibility Equation (4), and the values of *f*_3_ and *f*_5_ are obtained. Based on the principle of superposition:

(10) $$f_{\mathrm{II}}={\left[\begin{array}{cccc} f_{1} & f_{2} & \cdots & f_{8} \end{array}\right]}^{T}={G_{I}}^{-1}\left[\begin{array}{c} S_{3}\\ S_{O3} \end{array}\right]f_{3}+{G_{I}}^{-1}\left[\begin{array}{c} S_{5}\\ S_{O5} \end{array}\right]f_{5}+{G_{I}}^{-1}F=\sum_{i=3,5}{G_{I}}^{-1}\left[\begin{array}{c} S_{i}\\ S_{\mathrm{Oi}} \end{array}\right]f_{i}+{G_{I}}^{-1}F$$

where:

(11) $$f_{I}={\left[\begin{array}{cc} f_{3} & f_{5} \end{array}\right]}^{T},\;f_{\mathrm{II}}={\left[\begin{array}{ccccc} f_{1} & \cdots & f_{i} & \cdots & f_{8} \end{array}\right]}^{T},\;i\neq 3,5$$

Equation (10) can then be rewritten as:

(12) $${G_{I}}^{-1}G_{\mathrm{II}}f_{I}-f_{\mathrm{II}}+{G_{I}}^{-1}F=0$$

### 3.2. Establishment of the Measurement Model

Once the forces on the measurement modules are obtained:

(13) $$f={\left[\begin{array}{cc} {f_{I}}^{T} & {f_{\mathrm{II}}}^{T} \end{array}\right]}^{T}$$

Equations (4) and (12) can be obtained simultaneously as:

(14) $$\left[\begin{array}{cc} \delta & 0\\ {G_{\mathrm{sd}}}^{-1}G_{II} & -I \end{array}\right]f+\left[\begin{array}{c} \Delta_{F}\\ {G_{I}}^{-1}F \end{array}\right]=0$$

and then ***δ*** and $\Delta_{F}$ can be obtained simultaneously, thus yielding:

(15) $$\delta =\left[\begin{array}{cc} \sum_{j=1}^{8}\frac{{f_{j}}^{3}{f_{j}}^{3}l_{j}}{E_{j}A_{j}} & \sum_{j=1}^{8}\frac{{f_{j}}^{3}{f_{j}}^{5}l_{j}}{E_{j}A_{j}}\\ \sum_{j=1}^{8}\frac{{f_{j}}^{5}{f_{j}}^{3}l_{j}}{E_{j}A_{j}} & \sum_{j=1}^{8}\frac{{f_{j}}^{5}{f_{j}}^{5}l_{j}}{E_{j}A_{j}} \end{array}\right],\;\Delta_{F}=\left[\begin{array}{c} \sum_{j=1}^{8}\frac{{f_{j}}^{3}{f_{j}}^{t}l_{j}}{E_{j}A_{j}}\\ \sum_{j=1}^{8}\frac{{f_{j}}^{5}{f_{j}}^{t}l_{j}}{E_{j}A_{j}} \end{array}\right]$$

Then, the arrangement of the stiffness-relative items in Equation (15) can be rewritten as

(16) $$\delta ={\left[\begin{array}{cc} {f_{3}}^{3} & {f_{3}}^{5}\\ {f_{5}}^{3} & {f_{5}}^{5}\\ {f_{1}}^{3} & {f_{1}}^{5}\\ \vdots & \vdots \\ {f_{8}}^{3} & {f_{8}}^{5} \end{array}\right]}^{T}K^{-1}\left[\begin{array}{cc} {f_{3}}^{3} & {f_{3}}^{5}\\ {f_{5}}^{3} & {f_{5}}^{5}\\ {f_{1}}^{3} & {f_{1}}^{5}\\ \vdots & \vdots \\ {f_{8}}^{3} & {f_{8}}^{5} \end{array}\right],\;\Delta_{F}={\left[\begin{array}{cc} {f_{3}}^{3} & {f_{3}}^{5}\\ {f_{5}}^{3} & {f_{5}}^{5}\\ {f_{1}}^{3} & {f_{1}}^{5}\\ \vdots & \vdots \\ {f_{8}}^{3} & {f_{8}}^{5} \end{array}\right]}^{T}K^{-1}\left[\begin{array}{c} {f_{3}}^{t}\\ {f_{5}}^{t}\\ {f_{1}}^{t}\\ \vdots \\ {f_{8}}^{t} \end{array}\right]$$

When the diagonal matrices of the form

(17) $$K^{-1}=\left[\begin{array}{ccc} \frac{l_{3}}{E_{3}A_{3}} & & \\ & \ddots & \\ & & \frac{l_{8}}{E_{8}A_{8}} \end{array}\right]$$

are expressed as:

(18) $${K_{1}}^{-1}=\left[\begin{array}{cc} \frac{l_{3}}{E_{3}A_{3}} & 0\\ 0 & \frac{l_{5}}{E_{5}A_{5}} \end{array}\right]\;\mathrm{and}\;{K_{2}}^{-1}=\left[\begin{array}{ccc} \frac{l_{1}}{E_{1}A_{1}} & & \\ & \ddots & \\ & & \frac{l_{8}}{E_{8}A_{8}} \end{array}\right]$$

and then the values of ***δ*** and $\Delta_{F}$ can be obtained by:

(19) $$\delta ={\left[\begin{array}{c} I\\ {G_{I}}^{-1}G_{II} \end{array}\right]}^{T}K^{-1}\left[\begin{array}{c} I\\ {G_{I}}^{-1}G_{II} \end{array}\right]={K_{1}}^{-1}+{\left({G_{I}}^{-1}G_{II}\right)}^{T}{K_{2}}^{-1}\left({G_{I}}^{-1}G_{II}\right)$$

(20) $$\Delta_{F}={\left[\begin{array}{c} I\\ {G_{I}}^{-1}G_{II} \end{array}\right]}^{T}K^{-1}\left[\begin{array}{c} 0\\ {G_{I}}^{-1}F \end{array}\right]={\left({G_{I}}^{-1}G_{II}\right)}^{T}{K_{2}}^{-1}\left({G_{I}}^{-1}F\right)$$

and substituting ***δ*** and $\Delta_{F}$ into Equation (12) yields:

(21) $$F=-G^{\prime }\left[\begin{array}{cc} {K_{1}}^{-1}+{\left({G_{I}}^{-1}G_{II}\right)}^{T}{K_{2}}^{-1}\left({G_{I}}^{-1}G_{II}\right) & 0\\ {G_{I}}^{-1}G_{II} & -I \end{array}\right]f$$

where:

(22) $$G^{\prime }=G_{I}{\left[\begin{array}{c} {\left({G_{I}}^{-1}G_{II}\right)}^{T}{K_{2}}^{-1}\\ I \end{array}\right]}^{-1}$$

### 3.3. Analysis of Friction Influence

**Figure 5:**
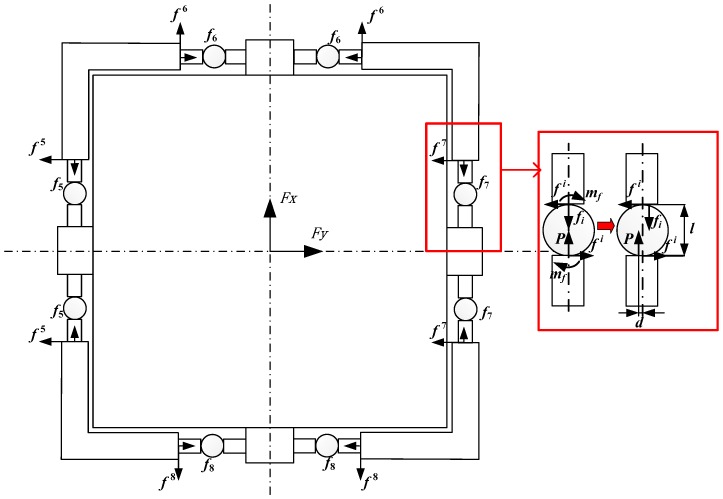
Force diagram of the proposed sensor in the X-Y plane.

When the measurement model is established using the force method, it is necessary to neglect the friction between branches. Although the steel ball structure is adopted for the proposed sensor to reduce the friction coupling, friction is unavoidable and must still be accounted for. The friction analysis of each measurement module in the proposed system is shown in Figure 5.

In Figure 5, the values of *f*^5^–*f*^8^ (the unified expression is *f^i^*) indicate the friction force between the measurement modules and platform, *f*_5_–*f*_6_ (the unified expression is *f_i_*) indicate the force transferred between the platform and measurement modules, *m_fi_* represents the rolling friction of the measurement modules, and *P* represents the force between the spoke force sensor and measurement modules. From Figure 5, the rolling friction on a steel ball measurement module is:

(23) $$f^{i}l=m_{fi}$$

and rolling friction is defined as:

(24) $$m_{fi}=Pd$$

The rearrangement of Equation (23) and its combination with Equation (24) yields:

(25) $$f^{i}=\frac{Pd}{l}$$

and because, by definition, *P* = *f_i_*:

(26) $$f^{i}=\frac{f_{i}d}{l}$$

Equation (26) is also applicable to vertical measurement modules; therefore, ***f***’ can be described by:

(27) $$f^{\prime }=\left[\begin{array}{c} f^{1}\\ f^{2}\\ \vdots \\ f^{8} \end{array}\right]=\left[\begin{array}{cccc} \frac{d_{1}}{l} & 0 & \cdots & 0\\ 0 & \frac{d_{2}}{l} & \cdots & 0\\ \vdots & \vdots & \ddots & \vdots \\ 0 & 0 & \cdots & \frac{d_{8}}{l} \end{array}\right]\left[\begin{array}{c} f_{1}\\ f_{2}\\ \vdots \\ f_{8} \end{array}\right]=A_{1}\left[\begin{array}{c} f_{1}\\ f_{2}\\ \vdots \\ f_{8} \end{array}\right]=A_{1}f$$

Here, ***f*’** represents the matrix of the frictional forces of measurement modules and ***f*** represents the matrix of the force transferred between the platform and the measurement modules. Moreover, $m_{f}$ = diag(*m_fi_*) can be obtained through Equation (23): $m_{f}=f^{\prime }l$.

According to the principle of virtual work:

(28) $$F^{T}V=f^{T}\overset{•}{q}+{\left(f^{\prime }\right)}^{T}{\overset{•}{\theta }}_{i}^{r}l_{x}+{\left(m_{f}\right)}^{T}{\overset{•}{\theta }}_{i}^{r}$$

where *i* denotes the *i*-th measurement module (a steel ball is equivalent to three rotational joints, and the axis of the third equivalent rotational joint coincides with the axial direction of the measurement module; thus, the load tangent to the rotation is smaller than those along the other axes; therefore, the friction force can be neglected here), *l_x_* represents the distance between the rotational joints and the centre of the rotation (based on the characteristics of the steel ball, this value is 0):

(29) $$V={\left[\begin{array}{cccccc} v_{x} & v_{y} & v_{z} & w_{x} & w_{y} & w_{z} \end{array}\right]}^{T}$$

is the generalized velocity for the upper platform:

(30) $$\overset{•}{q}={\left[\begin{array}{cccc} \overset{•}{q^{1}} & \overset{•}{q^{2}} & \cdots & \overset{•}{q^{8}} \end{array}\right]}^{T}$$

is the moving input speed of each measurement module, and:

(31) $${\overset{•}{\theta }}_{i}^{r}=J_{q}^{f}\overset{•}{q}$$

is the *r*-th joint velocity of the *i*-th measurement module.

According to Equation (23) and the mapping relation between the measurement module and upper platform of the sensor:

(32) $$F^{T}J_{q}^{p}\overset{•}{q}=f^{T}\overset{•}{q}+{\left(f^{\prime }\right)}^{T}{\overset{•}{\theta }}_{i}^{r}l$$

Once the same factors on both sides of Equation (31) are eliminated:

(33) $$F={\left[J_{P}^{q}\right]}^{T}f+{\left[J_{P}^{q}\right]}^{T}{\left[J_{q}^{f}\right]}^{T}f^{\prime }l$$

From Equations (23)–(27), the relationship between the frictional force, rolling friction, and axial force of the measurement branch can be found by substituting Equations (23)–(27) into Equation (33), yielding:

(34) F=Gf+Af

where:

(35) $$A={\mathrm{GG}}_{q}^{f}A_{1}l$$

and:

(36) $$G={\left[J_{P}^{q}\right]}^{T}$$

## 4. Results of Numerical Example

**Figure 6:**
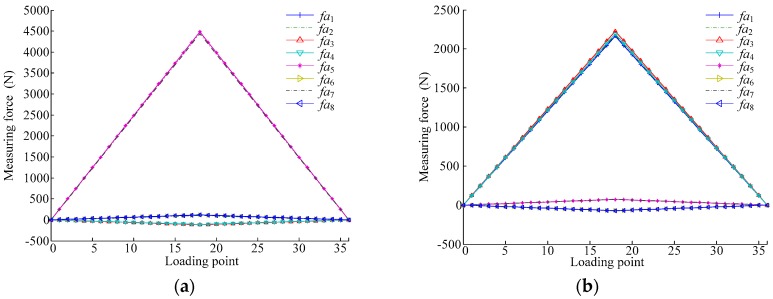
Output values of numerical model for load on the sensor under (**a**) *F_x_* and (**b**) *F_z_*.

Once the parameters of the physical sensor size (the length and width of the six-dimensional force sensor are 1.2 m, the distance between two adjacent vertical measurement modules is 1 m) and the value of the sensor axial stiffness obtained through calculation (2 × 10^8^ m/N baseline, varied by ±0.2 × 10^8^ m/N to account for small stiffness differences between modules) are inserted into the equations derived earlier, the force of each measurement module can be calculated. By using these force calculations, the expected response of each measurement module can be verified. The ideal curves produced by the measurement model of the proposed over-constrained parallel six-dimensional force sensor for each module output are shown in Figure 6, in which the generalized external force increases by 500 N at every loading point from 0 N (Point 0) to 9000 N (Point 18), and then decreases by 500 N at every loading point to 0 N (Point 36). Figure 6a shows the output curve when the sensor is subjected to a generalized external force in the X-direction, Figure 6b shows the output curve when the sensor is subjected to a generalized external force in the Z-direction.

The minimum and maximum output values of the numerical model for the measurement modules in the X-direction, shown in Figure 6a, are provided in Table 4.

The minimum and maximum output values of the numerical model for the measurement modules in the Z-direction, shown in Figure 6b, are listed in Table 5.

Table 4 and Table 5 illustrate that small differences between measurement module stiffnesses will inevitably lead to small differences in the output values of the modules for the proposed sensor.

**Figure 7:**
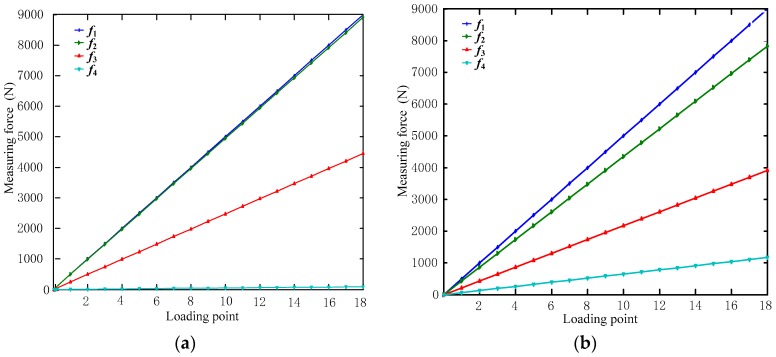
Output values of a measurement module accounting for (**a**) rolling friction and (**b**) sliding friction.

Next, the effect of both rolling and sliding friction on the measurement modules was determined and compared. By using Equation (34), the measured values of a sensor, accounting for rolling and sliding frictions, were determined (Figure 7).

**Figure 8:**
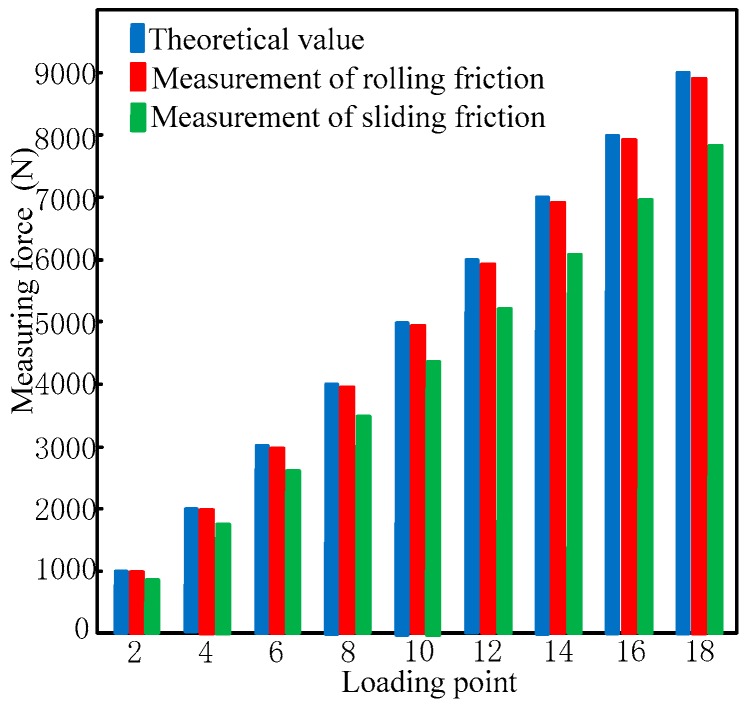
Comparison of calculated sensor loads accounting for various frictional forces.

In Figure 7, *f*_1_ indicates the measured output values without friction, *f*_2_ indicates the measured output values under (a) rolling friction and (b) sliding friction, *f*_3_ indicates the output values for a single measurement module in the measurement direction accounting for the frictions, and *f*_4_ indicates the output values of the frictions. The comparison of the output values in Figure 7 is shown in Figure 8.

Figure 7 and Figure 8 show that in an over-constrained six-dimensional sensor, friction is significant enough to influence the calculated output, with sliding friction having a much larger impact on the measured force than rolling friction. As a result, it is clear that the use of a steel ball structure, which eliminates sliding friction and thus the dimension coupling of the output, can contribute significantly to more accurate measurements.

## 5. Experimental Results

An experimental program was conducted to verify the performance of the proposed six-dimensional sensor. First, a calibration experiment was performed by applying a known load in given increments across the sensor to determine the error of the sensor and duplicate the results of the numerical example (the results shows that small differences exists among the output values of the modules in the proposed sensor). Then, a series of application experiments were conducted to confirm the ability of the proposed sensor to correctly detect the magnitude and distribution of the applied force.

### 5.1. Results of Calibration Experiment

**Figure 9:**
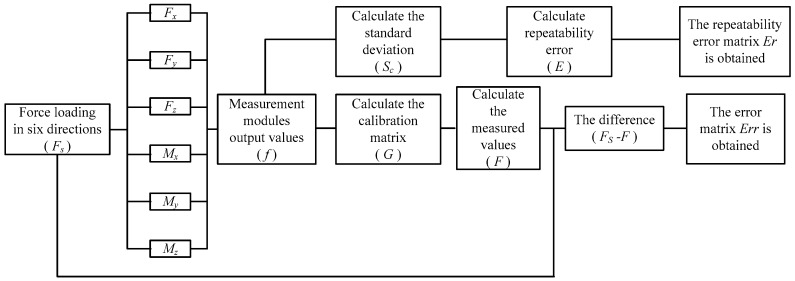
Flow chart of calibration experiment.

Figure 9 shows the process of the calibration experiment, in which a generalized external force (*F_S_*) was applied in six directions (loads were repeated three times in each direction) and increased by 500 N at every point from 0 N at point 0 to 9000 N at point 18, and then decreased by 500 N every point to 0 N at point 36. The output values (*f*) of the measurement modules could then be obtained to calculate the calibration matrix G, thus allowing the calculation of the measurement values (*F*) of the sensor; finally, the error matrix of the sensor was obtained through calculation. At the same time, the output values (*f*) of the measurement modules could then be obtained which is loaded three times in each direction to calculate the standard deviation, finally, the repeatability error matrix Er of the sensor was obtained through calculation.

**Figure 10:**
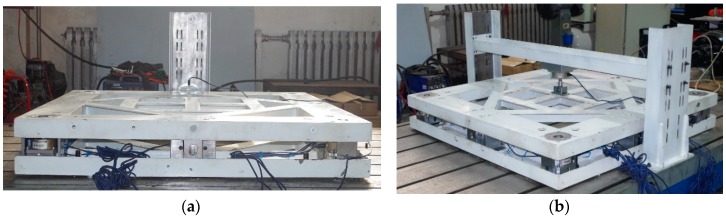
Calibration experiment setup for force loading of the proposed six-dimensional sensor in (**a**) the X-direction and (**b**) the Z-direction.

Figure 10 shows the calibration experiment set up.

**Figure 11:**
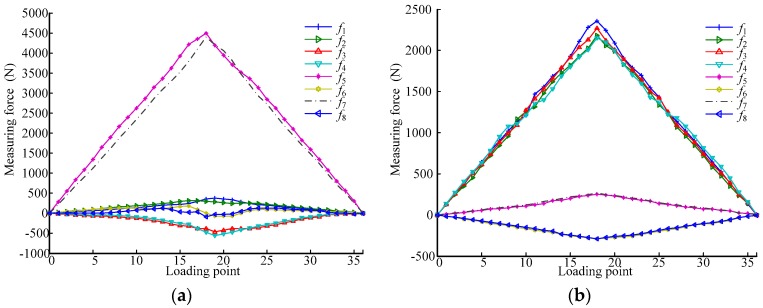
Output values of calibration experiment for load on the sensor under (**a**) *F_x_* and (**b**) *F_z_*.

Figure 11 shows the output values of each measurement module resulting from the calibration tests.

Table 6 lists the comparison of the maximum output values for the measurement modules in Figure 6a and Figure 11a.

In Table 6, *S*_1_ represents the standard deviation between *f*_5_ and *f*_7_ and *S*_2_ represents the standard deviation between *fa*_5_ and *fa*_7_. Table 7 shows the comparison of the maximum output values for the measurement module in Figure 6b and Figure 11b.

In Table 7, *S*_3_ represents the standard deviation between *f*_1_ to *f*_4_ and *S*_4_ represents the standard deviation between *fa*_1_ and *fa*_4_. As can be seen in Table 6 and Table 7, the differences between the values measured by the modules in the calibration experiment are slightly larger than those provided by the numerical calculation. The standard deviations *S*_1_, *S*_2_, *S*_3_, and *S*_4_ show that the deviation of the values measured by the modules in the calibration experiment from the average is larger than that in the numerical calculation.

In an ideal model, when the sensor is subjected to the maximum force *F_z_* in the Z-direction, the measurement modules 1–4 reach their maximal measuring range, and the output values of the four modules are equal. However, the force of each measurement module is uneven in the actual experiment, that is, when the sensor is subjected to maximum force *F_z_*, the output values of modules are not equal; some modules exceed their maximum range. Therefore, the actual maximum range of the sensor is less than *F_z_*. Here, if this property is called the ‘limit range’, it can be judged according to the standard deviation values. For example, in Table 6 and Table 7, *S*_3_ > *S*_1_; thus, the Z-direction range of the six-dimensional force sensor is more limited by the limit range.

In addition, it was noticed that this phenomenon affects the force-mapping matrix of the sensor; therefore, the force-mapping matrix of the sensor must be obtained through calibration experiments; such a force-mapping matrix is generally called calibration matrix. Based on the experimental data obtained through the calibration experiment, the calibration matrix was obtained using the least square method:

$$G=\left[\begin{array}{cccccccc} 2.6818 & -2.4136 & 2.4588 & -2.5201 & 1.8393 & 0.5275 & 0.1164 & -0.6579\\ 0.1737 & -0.1687 & 0.1633 & -0.1364 & -0.3648 & 1.0136 & 0.3442 & 1.0233\\ 1.7739 & 0.2602 & 1.6592 & 0.2632 & -0.2890 & 0.0385 & 0.3247 & -0.0105\\ 0.0114 & -3.9411 & 3.7468 & 0.0390 & -0.1984 & 0.4066 & 0.8001 & 0.1542\\ -1.7410 & 1.8484 & 2.1382 & -2.2391 & -0.3196 & -0.0282 & 0.3367 & 0.0294\\ 2.7510 & -2.6067 & 2.7684 & -2.6998 & 0.8618 & 1.9437 & -0.9323 & -2.1445 \end{array}\right]$$

The error matrix is calculated by:

(37) $$Err=\frac{\left|F_{S}-F\right|}{F_{FS}}$$

where *F_FS_* is the full scale (the full scales of *F_x_*, *F_y_*, and *F_z_* are 9000 N; the full scale of *M_x_*, *M_y_*, and *M_z_* are 9000 N·m) of the measurement direction, *F_S_* is the actual applied force/torque matrix, and *F* is the force/torque matrix calculated using the calibration matrix. The resulting error matrix is:

$$Err=\left[\begin{array}{cccccc} 0.0043 & 0.0098 & 0.0074 & 0.0068 & 0.0088 & 0.0198\\ 0.0037 & 0.0031 & 0.0021 & 0.0078 & 0.0078 & 0.0088\\ 0.0052 & 0.0043 & 0.0018 & 0.0083 & 0.0080 & 0.0034\\ 0.0033 & 0.0054 & 0.0026 & 0.0068 & 0.0082 & 0.0122\\ 0.0041 & 0.0023 & 0.0010 & 0.0080 & 0.0080 & 0.0128\\ 0.0053 & 0.0042 & 0.0078 & 0.0150 & 0.0150 & 0.0128 \end{array}\right]$$

**Figure 12:**
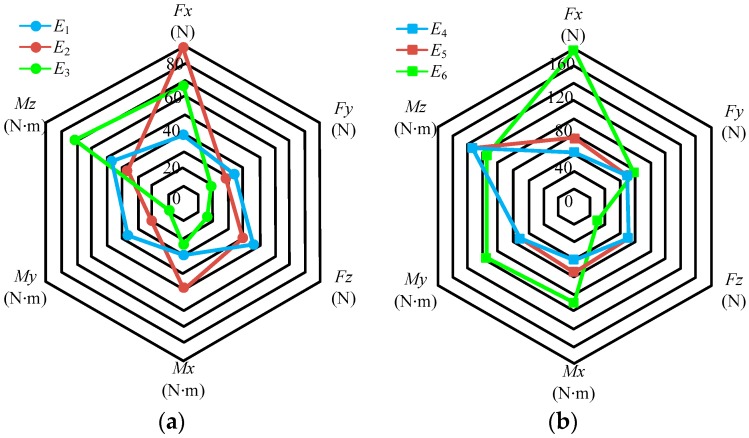
Error values in different directions of the sensor; (**a**) error output in loading directions of *F_x_*, *F_y_*, and *F_z_*; (**b**) error output in loading directions of *M_x_*, *M_y_*, and *M_z_*.

The force error values can be obtained by multiplying each element in the matrix *Err* with the full scale of the sensor; the results are shown in Figure 12.

In Figure 12, *E_i_* (*i* = 1, …, 6) represents the force error values, and is the product of *F_FS_* and the *i*-th column elements in matrix *Err*. The elements in the matrix *Err* imply the following: different rows represent the errors in different directions; thus, the first to sixth rows represent the directions of *F_x_*, *F_y_*, *F_z_*, *M_x_*, *M_y_*, and *M_z_*, respectively; different columns represent the different loading directions; thus, the first to sixth columns represent the loading directions of *F_x_*, *F_y_*, *F_z_*, *M_x_*, *M_y_*, and *M_z_*, respectively. For example, the element (0.0037) in the first column and second row represents the error in the *F_y_* direction when loaded in the *F_x_* direction. Therefore, the diagonal elements are represented as Class I errors, indicating the error between the measured and actual values; other elements are represented as Class II errors, indicating the error of the coupling output.

Class I errors for each dimension are as follows: *F_x_* (0.43%), *F_y_* (0.31%), *F_z_* (0.18%), *M_x_* (0.68%), *M_y_* (0.8%), and *M_z_* (1.28%). The maximum error of Class I type is 1.28% in *M_z_* and the maximum error of Class II type is 1.98% in *F_x_* when loaded by *M_z_*.

Repeatability is an important performance index of a six-dimensional force sensor; standard deviation is necessary to calculate repeatability. Based on the calculation of loads three times in each direction, the output values of measurement modules can be obtained and defined as fi=[fjki]j×k, where *i* = 1, 2, 3 represents the number of load times, *j* represents the number of measurement modules, *k* represents the number of loaded points. For the designed sensor, *j* = 8 and *k* = 35 (not included in the point at which the load value is 0). Then, the standard deviation matrix of the output values can be derived and expressed as *S_C_*, and *S_jk_* represents the elements in the matrix *S_C_*:

(38) Sjk=∑i=13(fjki−f¯jk)3−1

where ${\bar{f}}_{jk}$ represents the arithmetic mean values.

The standard uncertainty can be obtained:

$$U(A)=\frac{S_{c}}{\sqrt{3}}=\left[\begin{array}{cccccc} 24.440 & 15.695 & 21.548 & 9.0185 & 11.051 & 44.170\\ 5.7735 & 64.143 & 7.8102 & 13.203 & 15.099 & 14.525\\ 7.7674 & 4.7258 & 48.041 & 15.011 & 8.8889 & 7.0237\\ 5.8594 & 7.7674 & 5.2915 & 19.218 & 3.5118 & 11.060\\ 5.5075 & 5.1316 & 4.5092 & 8.1445 & 23.259 & 10.440\\ 8.1853 & 8.3864 & 12.897 & 20.526 & 18.147 & 14.571 \end{array}\right]$$

The formula for calculating the repeatability of a sensor is:

(39) $$E=\frac{kS_{c}}{F_{FS}}\times 100\%$$

where *k* represents the confidence coefficient; for the repeatability of a sensor, the general value of *k* is 2 or 3. Finally, the repeatability error matrix can be obtained according to the repeatability formula of the sensor:

$$Er=\left[\begin{array}{cccccc} 0.0094 & 0.0060 & 0.0083 & 0.0035 & 0.0042 & 0.0170\\ 0.0022 & 0.0037 & 0.0030 & 0.0051 & 0.0058 & 0.0056\\ 0.0030 & 0.0018 & 0.0185 & 0.0058 & 0.0034 & 0.0027\\ 0.0023 & 0.0030 & 0.0020 & 0.0074 & 0.0014 & 0.0042\\ 0.0021 & 0.0020 & 0.0017 & 0.0031 & 0.0090 & 0.0040\\ 0.0032 & 0.0032 & 0.0050 & 0.0079 & 0.0070 & 0.0056 \end{array}\right]$$

**Figure 13:**
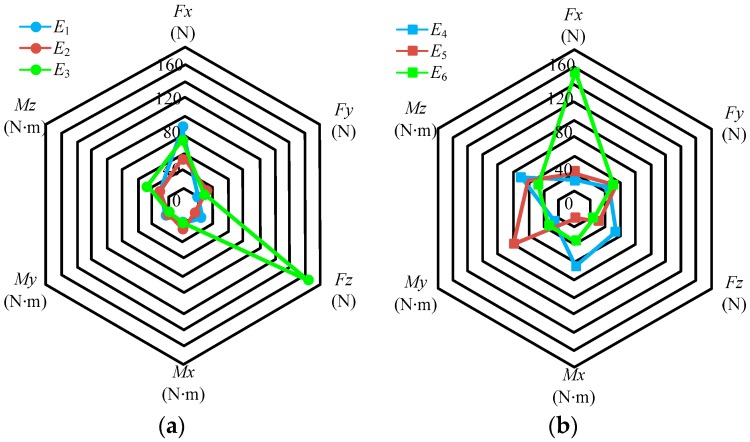
Repeatability error values in different directions of the sensor; (**a**) error output in loading direction of *F_x_*, *F_y_*, and *F_z_*; (**b**) error output in loading direction of *M_x_*, *M_y_*, and *M_z_*.

Note that according to the expression forms of the six-dimensional force sensor, the elements in matrix ***E****r* are not multiplied by the percentage. For matrix ***E****r*, the diagonal elements represent the repeatability error of the measurement values, and other elements represent the repeatability error of the coupling output. For example, the element (0.0094) in the first column and first row represents the repeatability error in the *F_x_* direction when the loading is perfromed in the *F_x_* direction three times; the element (0.0022) in the first column and second row represents the repeatability error in the *F_y_* direction when loading is performed in the *F_x_* direction three times. The force repeatability error values can be obtained through multiplying each element in matrix ***E****r* with the full scale of the sensor; the results are shown in Figure 13. In Figure 13, *E_i_* (*i* = 1, …, 6) represents the repeatability force error values; it is the product of *F_FS_* and the *i*-th column elements in matrix ***E****r*.

The repeatability errors for measurement values of each dimension are as follows: *F_x_* (0.94%), *F_y_* (0.37%), *F_z_* (1.85%), *M_x_* (0.74%), *M_y_* (0.9%), and *M_z_* (0.56%). The maximum repeatability error of the measurement values is 1.85% in *F_z_* and the maximum repeatability error of the coupling output is 1.7% in *F_x_* when loaded by *M_z_*.

### 5.2. Results of Application Experiments

**Figure 14:**
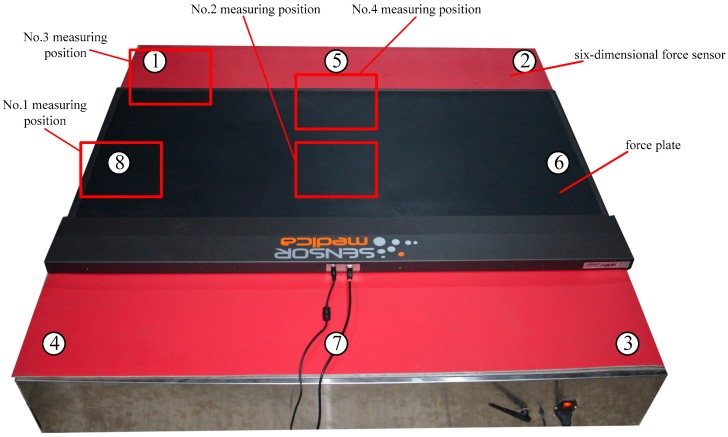
Measuring positions for the application experiments using a force plate and the proposed six-dimensional force sensor.

To test the application of the proposed six-dimensional force sensor, a force plate was used in conjunction with the proposed sensor, shown in Figure 14. The force plate is capable of measuring the plantar pressure distribution and force area of the feet standing upon it; thus, by placing the force plate on the proposed six-dimensional force sensor, the Z-direction of the force can be measured by the sensor and correlated with the measurement of the force plate. The combined use of the force plate and sensor can measure the force distribution of a standing human, allowing the determination of the sensor’s performance and verification of the accuracy of the sensor measurements.

**Figure 15:**
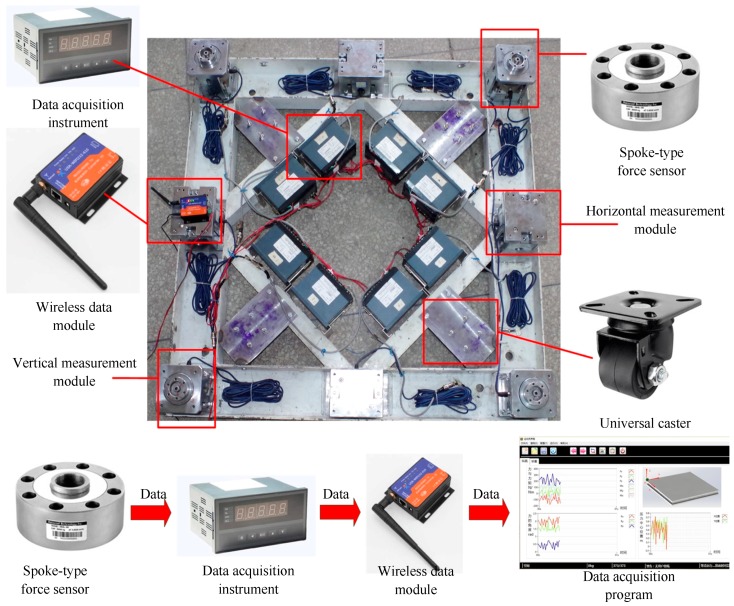
Internal components of the completed proposed six-dimensional force sensor used in the application experiments.

Figure 14 shows the completely assembled six-dimensional force sensor, which is protected by stainless steel plates all around it. The labels for each measurement module contained by the sensor are indicated by the circled numbers in the figure. Label Nos. 1–4 indicate the different positions at which a standing human was measured. The internal components of the completed proposed six-dimensional force sensor are shown in Figure 15.

In Figure 15, the spoke-type force sensors in the vertical and horizontal measuring modules output their signals to the data acquisition instrument, and then the data is collected and transmitted to an external computer via a wireless data module. The universal caster installed on the lower platform allows the sensors to move freely.

**Figure 16:**
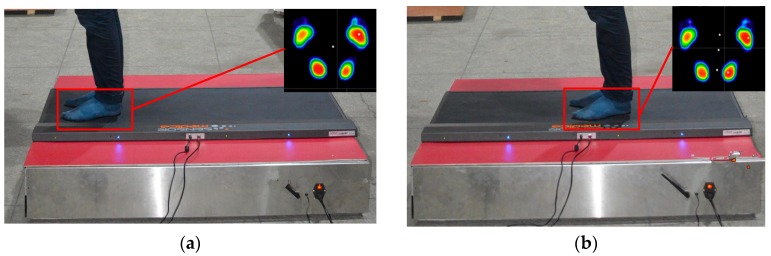

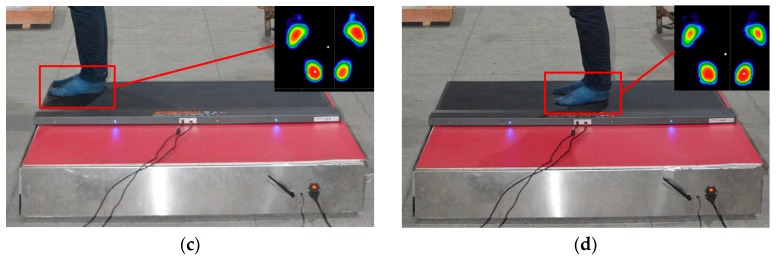
Force plate measurements with person standing at (**a**) Position 1; (**b**) Position 2; (**c**) Position 3; and (**d**) Position 4.

**Figure 17:**
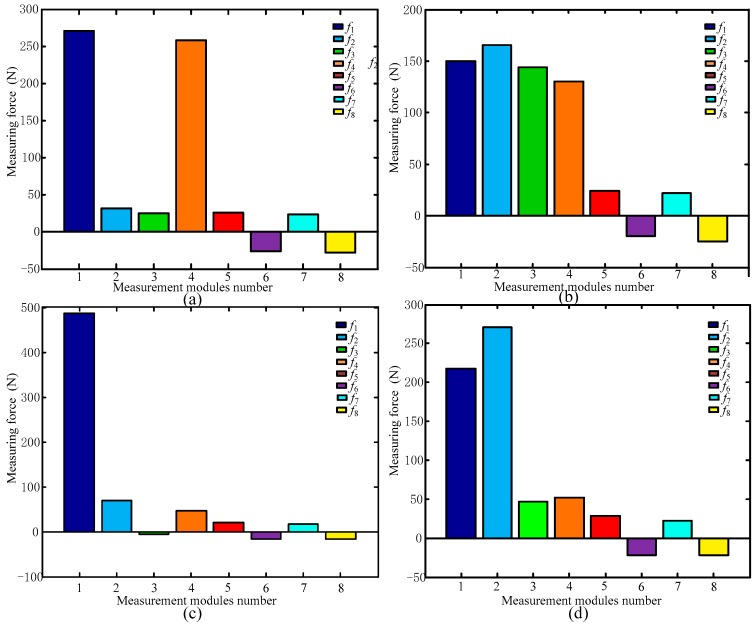
Six-dimensional sensor measurement module outputs with a person standing at (**a**) Position 1; (**b**) Position 2; (**c**) Position 3; and (**d**) Position 4.

The sensor tests performed with a person standing in different positions, as indicated in Figure 14, are shown in Figure 16, with the plantar pressure distribution measured using the force plate shown in the upper right inset of each figure. The corresponding output values for the measurement modules of the six-dimensional sensor are shown in Figure 17.

**Figure 18:**
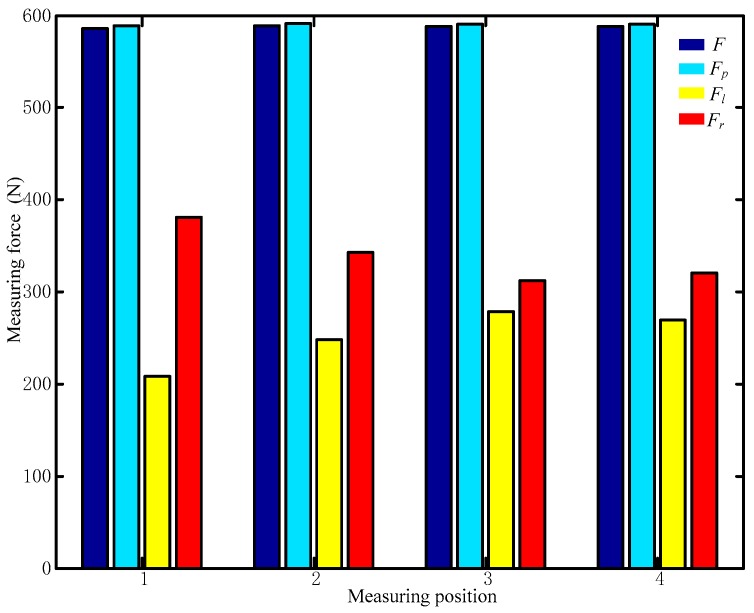
Comparison of values measured by the force plate and proposed six-dimensional sensor in application experiments.

Table 8 shows the output values of each measurement module at each position, and Figure 18 shows the comparison of the values measured by the force place and proposed six-dimensional sensor.

The measured values of the proposed sensor and force plate are shown in Figure 18 and Table 9, in which *F* is the measurement of the proposed sensor, *F_p_* is the measured value of the force plate, *F_r_* is the force of the right foot measured using the force plate, *F_l_* is the force of the left foot measured using the force plate.

As shown in Figure 17 and Figure 18, while the values measured by each measurement module are different for different standing positions, the total force measured by the proposed six-dimensional sensor is close to the value measured by the force plate for each position. The differences between measured values for Positions 1–4 are 3.05, 1.89, 2.78, and 2.64 N, respectively. While this confirms that the proposed six-dimensional force sensor can be used to measure the position of a generalized external force, the analysis and experiment show that when a person is standing in the centre of the sensor (for example, Position 2 shown in Figure 16b), the values measured by each measurement module are different; therefore, the results of the aforementioned calibration experiment must be applied to reduce this error.

**Figure 19:**
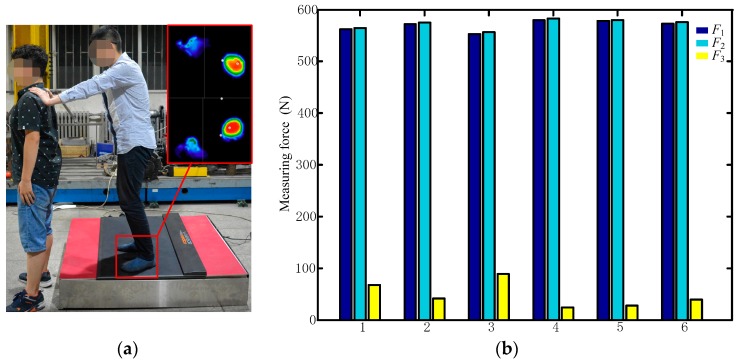
Testing the force distribution when a standing person pushes on another person: (**a**) experimental conditions. (**b**) Comparison of measured values from the force plate and proposed sensor under these conditions.

When measuring Position 1, the force plate indicates that the pressure on the right foot is greater than that on the left foot, and the values measured by the proposed sensor show that Module 1 is reporting a greater load than Module 4, indicating that the sensor reflects the conditions measured by the force plate. Position 2 shows a similar pattern. To apply a more concentrated load, the person standing on the sensor assembly pushed on another person in the manner shown in Figure 19a. In this case, the experiment recorded data from the proposed sensor and force plate at six force values, with the results shown in Figure 19b and Table 10, in which *F*_1_ is the vertical force measured by the proposed sensor, *F*_2_ is the vertical force measured by the force plate, and *F*_3_ is the horizontal force measured by the proposed sensor.

Note that the force plate can only measure the plantar pressure in the vertical direction, in which the proposed sensor produces measured values within 0.57% of those reported by the force plate. The forces measured by the proposed sensor in the horizontal direction, which cannot be compared to data obtained from the force plate, are 68, 42, 89, 24, 28, and 39 N at Points 1–6, respectively.

## 6. Conclusions

An over-constrained six-dimensional force sensor was proposed using novel measurement modules, which utilize steel ball structures to measure force in all six-dimensions. In addition, the measurement modules can accommodate both tension and pressure while remaining compact.

Based on the characteristics of the proposed force sensor, the stiffness difference of each measurement module was considered, and static equilibrium and displacement compatibility equations were established to obtain a transformation function. By using the virtual work principle, the influence of friction on the measurement accuracy of the sensor was then analysed. Next, the measurement characteristics of the proposed sensor were analysed through the derived numerical examples.

Finally, calibration and application experiments were performed on the proposed sensor. The measurement accuracy of the sensor was determined to be 1.28%, with a maximum coupling error of 1.98%. A force plate was used in conjunction with the proposed sensor, allowing application experiments to be conducted to statically measure the distribution of the plantar force of the human body. The results indicated that the value measured by the proposed sensor is close to the value measured by the force plate, with a difference within 0.57% between the measured values.

The proposed six-dimensional force sensor using steel ball measurement modules was demonstrated to be capable of accurately detecting the magnitude and distribution of loads equivalent to the weight of a human body. By using the steel ball structure, the effects of friction coupling were minimised, while the proposed measurement modules ensured that both pressure and tension could be measured in their given orientation. This sensor has the potential for application in fields such as human body biomechanics measurements. In subsequent studies, the proposed six-dimensional force sensor will be applied to human body biomechanics measurements. However, the acquisition frequency of the current data acquisition system reading the sensor is insufficient for human body dynamic forces, such as squatting and other movements. In subsequent studies, a new acquisition system will be adopted including both a force plate and high-speed camera in conjunction with the proposed sensor to conduct dynamic measurements of human biomechanics.

## Figures and Tables

**Table 1:** Labelled parts of the six-dimensional force sensor.

**Label**	**1**	**2**	**3**	**4**	**5**	**6**
**part**	vertical pre-tightening device	adjustment component	upper vertical branch module	steel ball	pressure-head	spoke-type force sensor
**Label**	**7**	**8**	**9**	**10**	**11**	**12**
**part**	mounting plate	lower vertical branch module	adjustment part	horizontal pre-tightening device	lower horizontal branch module	upper horizontal branch module

**Table 2:** The specifications of the major parts of the sensor.

Part	Upper Vertical Branch Module	Steel Ball	Spoke-Type Force Sensor	Lower Vertical Branch Module	Lower Horizontal Branch Module	Upper Horizontal Branch Module
**Length (mm)**	134	-	-	134	156	156
**Width (mm)**	134	-	-	134	134	134
**Height (mm)**	143	-	37	53.5	136	133
**Diameter (mm)**	-	20	105	-	-	-

**Table 3:** Cases for force *f_j_^i^* on the measurement modules.

Case	1	2	3	4	5
**Value**	1	0	${G_{I}}^{-1}\left[\begin{array}{c} S_{i}\\ S_{Oi} \end{array}\right]$	0	*G_I_*^−1^*F*
**Condition**	*j =* 3, 5, *i* = *j*	*j* = 3, 5, *i* ≠ *j*	*j* ≠ 3, 5, *i* = *3, 5*	*j* = 3, 5, *i* = *t*	*j* ≠ 3, 5, *i* = *t*

**Table 4:** Output value of generalized force in the X-direction.

Load Point	*fa*_5_ (N)	*fa*_7_ (N)
0	0	0
18	4487.4	4437.9
36	0	0

**Table 5:** Output value of generalized force in the Z-direction.

Load Point	*fa*_1_ (N)	*fa*_2_ (N)	*fa*_3_ (N)	*fa*_4_ (N)
0	0	0	0	0
18	2163.6	2180.7	2225.7	2193.3
36	0	0	0	0

**Table 6:** Calculated and experimental maximum measurement module output values.

Load Point	*f*_5_ (N)	*f*_7_ (N)	*S*_1_ (N)	*fa*_5_ (N)	*fa*_7_ (N)	*S*_2_ (N)
0	0	0	0	0	0	0
18	4499	4400	70	4487.4	4437.9	35
36	0	0	0	0	0	0

**Table 7:** Calculated and experimental maximum measurement module output values.

Load Point	*f*_1_ (N)	*f*_2_ (N)	*f*_3_ (N)	*f*_4_ (N)	*S*_3_(N)	*fa*_1_ (N)	*fa*_2_ (N)	*fa*_3_ (N)	*fa*_4_ (N)	*S*_4_(N)
0	0	0	0	0	0	0	0	0	0	0
18	2360	2183	2269	2150	94.1	2163.6	2180.7	2225.7	2193.3	26.2
36	0	0	0	0	0	0	0	0	0	0

**Table 8:** Output values of each measurement module in application experiments.

Position	*f*_1_ (N)	*f*_2_ (N)	*f*_3_ (N)	*f*_4_ (N)	*f*_5_ (N)	*f*_6_ (N)	*f*_7_ (N)	*f*_8_ (N)
1	271	32	25	258	26	−26	24	−28
2	150	186	133	120	24	−20	22	−25
3	479	69	−7	47	21	−17	16	−17
4	218	271	47	52	28	−22	22	−22

**Table 9:** Comparison of values measured by the force plate and proposed six-dimensional sensor in application experiments.

Position	*F* (N)	*F_p_* (N)	*F_l_* (N)	*F_r_* (N)
1	586	589.05	208.02	381.03
2	589	590.89	248.17	342.72
3	588	590.78	278.62	312.16
4	588	590.64	269.73	320.91

**Table 10:** Comparison of measured values from the force plate and proposed sensor when a standing person pushes on another.

Force	1	2	3	4	5	6
*F*_1_ (N)	562	572	551	580	578	573
*F*_2_ (N)	564.52	575.29	553.83	582.64	580.01	576.09
*Difference*	2.52	3.29	2.83	2.64	2.01	3.09

## References

[1] Watson P.C., Drake S.H. Pedestal Wrist Force Sensors for Industrial Assembly. Proceedings of the 5th International Symposium on Industrial Robots.

[2] Kroll E., Weill R. Decoupling Load Components and Improving Robot Interfacing with an Easy-to-use 6-axis Wrist Force Sensor. Proceedings of the 7th World Congress Theory on Machines and Mechanisms.

[3] Gao F., Zhang G.Y., Zhao X.C., Guo W.Z. The design and applications of F/T sensor based on Stewart platform. Proceedings of the 12th IFToMM World Congress.

[4] Gao F., Zhang J., Chen Y.L., Jin Z.L. Development of a new type of 6-DOF parallel micro-manipulator and its control system. Proceedings of the IEEE International Conference Robotic Intelligent System Signal Process.

[5] Liang Q., Zhang D., Wang Y. (2013). PM based multi-component F/T sensors—State of the art and trends. Robot. Comput. Integr. Manuf..

[6] Hirose S., Yoneda K. Robotic Sensors with Photo-detecting Technology. Proceedings of the 20th ISIR.

[7] Ranganath R., Nair P.S., Mruthyunjaya T.S. (2004). A Force-torque Sensor Based on a Stewart Platform in a Near-singular Configuration. Mech. Mach. Theory.

[8] Dwarakanath T.A., Dasgupta B., Mruthyunjaya T.S. (2001). Design and Development of a Stewart Platform Based Force-torque Sensor. Mechatronics.

[9] Dwarakanath T.A., Venkatesh D. (2006). Simply supported, ‘Joint less’ parallel mechanism based force-torque sensor. Mechatronics.

[10] Wright A.M., Wright A.B., Born T., Strickland R. (2013). A six degree-of-freedom thrust sensor for a lab scale hybrid rocket. Meas. Sci. Technol..

[11] Wang Z.J., Li Z.X., He J. (2013). Optimal design and experiment research of a fully pre-stressed six-axis force/torque sensor. Measurement.

[12] Wang Z.J., Yao J.T. (2012). Hyper static analysis of a fully pre-stressed six-axis force/torque sensor. Mech. Mach. Theory.

[13] Jia Z.Y., Lin S., Liu W. (2010). Measurement method of six-axis load sharing based on the Stewart platform. Measurement.

[14] Yao J.T., Cai D., Zhang H., Wang H., Wu D., Zhao Y.S. (2016). Task-oriented design method and research on force compliant experiment of six-axis wrist force sensor. Mechatronics.

[15] Pashikevich A., Chablat D., Wenger P. (2009). Stiffness Analysis of Overstrained Parallel Manipulators. Mech. Mach. Theory.

[16] Janus F., Marek W. (2011). On the Unique Solve ability of a Direct Dynamic Problem for Mechanism with Redundant Constraints and Coulomb Friction in Joints. Mech. Mach. Theory.

[17] Choi Y.J., Sreenivasan S.V., Choi B.J. (2008). Kinematic design of large displacement precision XY positioning stage by using cross strip flexure joints and over-constrained mechanism. Mech. Mach. Theory.

[18] Yao J.T., Zhang H.Y. (2015). Isotropy analysis of redundant parallel six-axis force sensor. Mech. Mach. Theory.

[19] Yao J.T., Zhu J.L., Wang Z.J. (2013). Measurement theory and experimental study of fault-tolerant fully pre-stressed parallel six-component force sensor. IEEE Sens. J..

[20] Dwarakanath T.A., Gaurav B. (2011). Beam type hexapod structure based six component force- torque sensor. Mechatronics.

[21] Zhao Y.S., Hou Y.L., Yan Z.W. Research and Design of a Pre-Stressed Six-Component Force/Torque Sensor Based on the Stewart Platform. Proceedings of the ASME 2005 International Design Engineering Technical Conferences and Computers and Information in Engineering Conference.

[22] Liang Q.K., Zhang D. (2011). Six-DOF micro-manipulator based on compliant parallel mechanism with integrated force sensor. Robot. Comput. Integr. Manuf..

[23] Liang Q.K., Zhang D., Coppola G., Mao J., Sun W., Wang Y., Ge Y. (2016). Design and Analysis of a Sensor System for Cutting Force Measurement in Machining Processes. Sensors.

[24] Zhao Y.Z., Jiao L.H., Weng D.C., Zhang D., Zheng R.C. (2016). Decoupling Principle Analysis and Development of Parallel Three-Dimensional Force Sensor. Sensors.

[25] Lu Y., Wang Y., Ye N., Chen L. (2017). Development of a novel sensor for hybrid hand with three fingers and analysis of its measure performances. Mech. Syst. Signal Process..

[26] Kim U., Lee D.H., Kim Y.B., Seok D.Y., Choi H.R. (2017). A Novel Six-Axis Force/Torque Sensor for Robotic Applications. IEEE-ASME Trans. Mech..

[27] Yang E.C.Y. (2016). Design and Sensitivity Analysis Simulation of a Novel 3D Force Sensor Based on a Parallel Mechanism. Sensors.

[28] Zhao Y.Z., Jiao L.H., Weng D.C., Zhang D., Zheng R.C. (2016). Mathematical Model and Calibration Experiment of a Large Measurement Range Flexible Joints 6-UPUR Six-Axis Force Sensor. Sensors.

